# Sequential Pathology of a Genotype XIII Newcastle Disease Virus from Bangladesh in Chickens on Experimental Infection

**DOI:** 10.3390/pathogens9070539

**Published:** 2020-07-06

**Authors:** Congriev Kumar Kabiraj, Tanjin Tamanna Mumu, Emdadul Haque Chowdhury, Mohammad Rafiqul Islam, Mohammed Nooruzzaman

**Affiliations:** Department of Pathology, Faculty of Veterinary Science, Bangladesh Agricultural University, Mymensingh 2202, Bangladesh; congriev@bau.edu.bd (C.K.K.); tanjinmumu@bau.edu.bd (T.T.M.); emdad@bau.edu.bd (E.H.C.); mrislam@bau.edu.bd (M.R.I.)

**Keywords:** newcastle disease virus, genotype XIII, velogenic pathotype, lymphoid depletion, meningoencephalitis, Bangladesh

## Abstract

The sequential pathology of a genotype XIII Bangladeshi strain of Newcastle disease virus (NDV) was studied in 5-weeks old chickens. Layer chickens of ISA Brown breed were inoculated through the intranasal and intraocular routes with the BD-C161/2010 strain of NDV and examined at different times post-infection (pi). NDV-infected chickens showed depression at 3 days pi (dpi) followed by dropped wings, paralysis and death starting at 4 dpi. Lungs of infected chickens showed hemorrhagic lesions starting at 24 hours pi (hpi) that was followed by pallor and slight contraction by 2 to 3 dpi and subsequently developed into severe hemorrhagic pneumonia with mononuclear cell infiltration. Hemorrhagic and necrotizing lesions were found in different visceral organs including proventriculus, intestine, gut-associated lymphoid tissues, liver and kidneys starting at 3 dpi that progressed rapidly. Severe lymphoid depletion was observed in the thymus, spleen and bursa of Fabricius starting at 1–3 dpi followed by hemorrhages, necrosis, inflammation and atrophy at 4–5 dpi. In the brain, mild neuronal lesions such as focal to diffuse encephalitis with encephalomalacia was observed at 2–3 dpi and moderate and diffuse meningoencephalitis with encephalomalacia at advanced stages. In conclusion, the BD-C161/2010 strain of NDV produced lesions typical of velogenic viscerotropic pathotype of NDV.

## 1. Introduction

Newcastle disease virus (NDV) is classified as virulent strains of avian paramyxovirus type 1 (APMV-1) serotype under the genus *Avian orthoavulavirus 1*, family *Paramyxoviridae* [1]. NDV is a non-segmented, single-stranded, negative-sense, enveloped RNA virus [2]. The genome of NDV is approximately 15.2 kilobases (kb) that encodes six structural proteins. Historically, NDV strains have been classified into 4 virulence groups: velogenic (high virulence, up to 100% mortality), mesogenic (moderate virulence with respiratory signs and lower level of mortality), lentogenic (low virulence with mild or inapparent respiratory signs) and avirulent (asymptomatic) based on the clinical disease they produced in infected chickens [3]. Velogenic strains are further divided into velogenic viscerotropic causing severe gastrointestinal and visceral hemorrhages, or neurotropic causing respiratory and neurologic clinical signs and encephalitis [3,4,5,6,7]. Although all NDV strains belong to a single serotype, there is large genetic diversity among NDV isolates. Two classes of NDV have been identified; viruses under Class I contains only one genotype with three sub-genotypes, whereas viruses of Class II are divided into at least 20 genotypes and many sub-genotypes [8]. 

Velogenic pathotype of NDV under genotype XIII is prevailing in Bangladesh causing substantial economic losses [9,10,11,12]. Virulent strains of NDV are defined by the World Organization for Animal Health (OIE) as viruses that have an intracerebral pathogenicity index (ICPI) of 0.7 or higher (2.0 is maximum) or a fusion protein cleavage site (FPCS) with multiple basic amino acids and phenylalanine at position 117 [13]. However, the clinical diseases produced by different pathotypes do not always mirror the ICPI. For example, there are cases in which an NDV strain is considered as virulent by ICPI but does not produce much severe clinical disease [6,14]. Similarly, NDV strains with a velogenic FPCS motif and a relatively higher ICPI (>1.5) show variable clinicopathological features and virus replication in tissues upon experimental infection in specific pathogen-free (SPF) chickens [6]. Moreover, the F protein is not the only determinant of NDV virulence [15,16]. A difference in the pathogenesis of NDV strains belonging to different genotypes has also been observed [6]. Therefore, full clinicopathological assessment in susceptible host species upon experimental infection is necessary for a more accurate description of the pathogenicity of a new NDV strain [13].

Although pathological changes have been well studied in ND affected dead or sick birds, very little is known about the progressive development of pathological changes in NDV-infected birds. Genotype XIII is a recently evolving genotype of NDV and an in-depth pathological study considering at close time points under experimental condition has not been performed yet. Here we analyzed the sequential pathological changes in chickens experimentally infected with a genotype XIII NDV strain from Bangladesh.

## 2. Results

### 2.1. Clinical Features

NDV infected chickens showed signs of depression at 3 days post-infection (dpi) which gradually became severe with ruffled feathers, dropped wings, paralysis and death starting at 4 dpi. Chickens in the control group remained normal throughout the study period.

### 2.2. Gross Lesions

**Figure 1:**
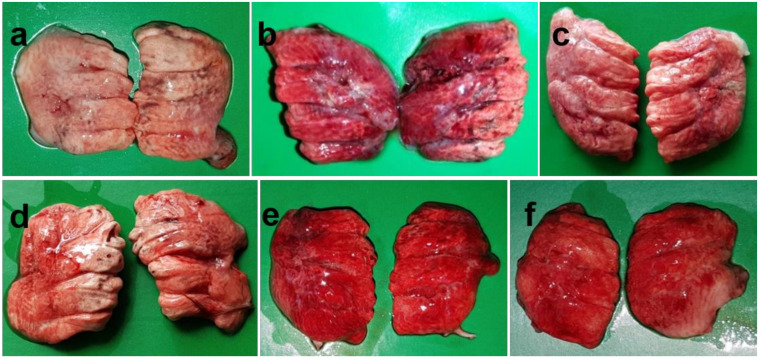
Gross pathological changes in lungs of NDV infected chickens. Lungs of control chickens showing normal appearance (**a**). Lungs of infected chickens showing congestion, hemorrhage and consolidation with traces of fibrin at 24 hpi (**b**), gradual paleness and reduction in size of lungs at 2 dpi (**c**) and 3 dpi (**d**), and severe congestion, hemorrhage, and gradual shrinking of lungs at 4 dpi (**e**) and 5 dpi (**f**).

The NDV infected chickens were examined at autopsy and gross pathological changes were recorded. Lesions in the lungs (Figure 1) started with congestion, hemorrhages and consolidation with traces of fibrin at 24 hpi.

**Figure 2:**
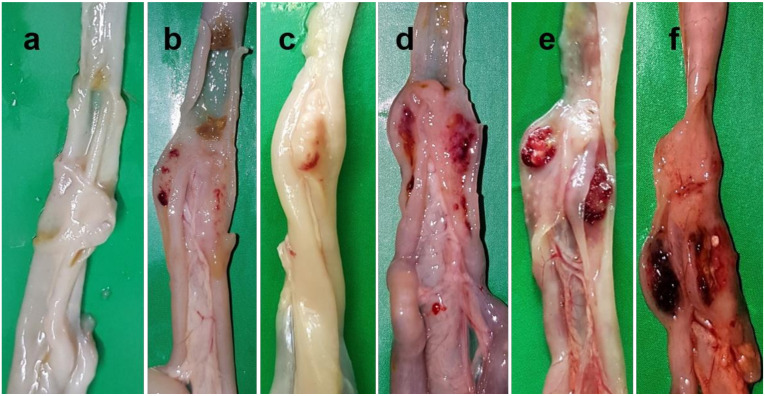
Gross pathological changes in cecal tonsils of NDV infected chickens. Cecal tonsils of control chickens showing normal appearance (**a**). Cecal tonsils of infected chickens showing ecchymotic hemorrhages at 24 hpi (**b**), coalescence of ecchymotic hemorrhages to hemorrhagic spot at 2 dpi (**c**), abundant hemorrhagic spots with increased sizes at 3 dpi (**d**), thickened, edematous, inflamed cecal tonsils with larger and numerous hemorrhagic spots at 4 dpi (**e**) and 5 dpi (**f**).

Gradually it became pale and somewhat reduced in size by 2–3 dpi. However, the lungs became severely hemorrhagic again after 3 days and continued till the end of the study. Cecal tonsils showed initial ecchymotic hemorrhages at 24 hpi, which gradually coalesced to form hemorrhagic spots at 2–3 dpi. With the progression of the disease, the hemorrhagic lesions became severe with larger and numerous hemorrhagic spots on thickened, edematous and inflamed cecal tonsils at 4–5 dpi (Figure 2).

**Figure 3:**
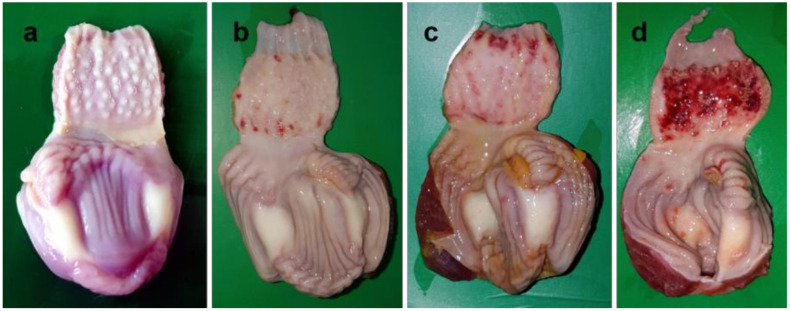
Gross pathological lesions in the proventriculus of chickens infected with NDV. (**a**) The proventriculus of control chickens showing normal appearance (**a**). The proventriculus of infected chickens showing several ecchymotic hemorrhages on the tip of the proventricular glands at 3 dpi (**b**), edema with increased number of solitary and coalesced ecchymotic hemorrhages on the proventricular glands at 4 dpi (**c**), and numerous hemorrhagic spots coalesced to form brush paint appearance on the entire mucosa at 5 dpi (**d**).

Lesion in the proventriculus was first observed at 3 dpi characterized by several ecchymotic hemorrhages on the tip of proventricular glands. With time, thickened mucosa with an increased number of solitary and coalesced ecchymotic hemorrhages on the proventricular glands was observed, which gradually converted to brush paint appearance and covered the entire mucosal surface at 5 dpi (Figure 3).

**Figure 4:**
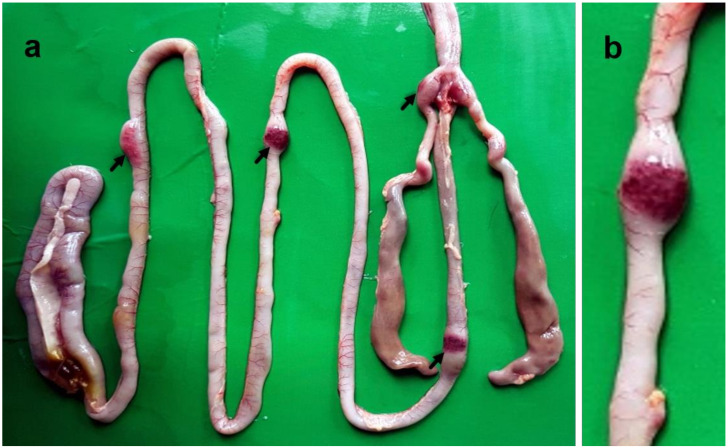
Gross pathological lesions in the intestine of chickens infected with NDV showing button-like ulcers characterized by multiple thickened, edematous and hemorrhagic gut-associated lymphoid tissues (GALT) (**a**). Zoomed photograph of the intestine showing a typical button-like ulcer (**b**).

In the intestine, button-like ulcers characterized by thickened, edematous and hemorrhagic lesions at multiple sites of gut-associated lymphoid tissues (GALT) appeared at 3 dpi, which increased in size and number with the progression of the disease (Figure 4). Other visceral organs such as the liver (Supplementary Figure S1) and kidneys (Figure not shown) showed mild congestion and hemorrhages at 3 dpi, which progressed rapidly with enlargement of the organ at an advanced stage of the disease.

**Figure 5:**
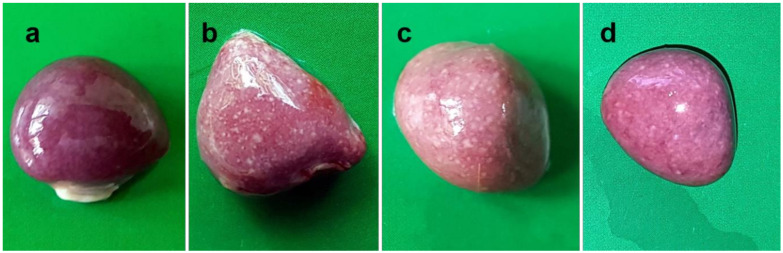
Gross pathological lesions in the spleen of NDV infected chickens. The spleen of control chickens showing normal appearance (**a**). Spleens of infected chickens showing numerous small necrotic foci at 3 dpi (**b**), numerous small to larger necrotic foci at 4 dpi (**c**), and numerous necrotic foci with atrophy of the organ at 5 dpi (**d**).

Among various lymphoid organs, the spleen showed numerous small white necrotic foci at 3 dpi. The number and size of the necrotic foci increased with time, which led to atrophy of the spleen at 5 dpi (Figure 5). Edematous bursa of Fabricius containing yellowish caseous mass was found during 2–3 dpi. Following that, the organ became atrophied and hemorrhagic at 4–5 dpi (Figure not shown). No abnormal changes were noticed in the uninfected chickens at any time points of the study.

### 2.3. Histopathological Changes

**Figure 6:**
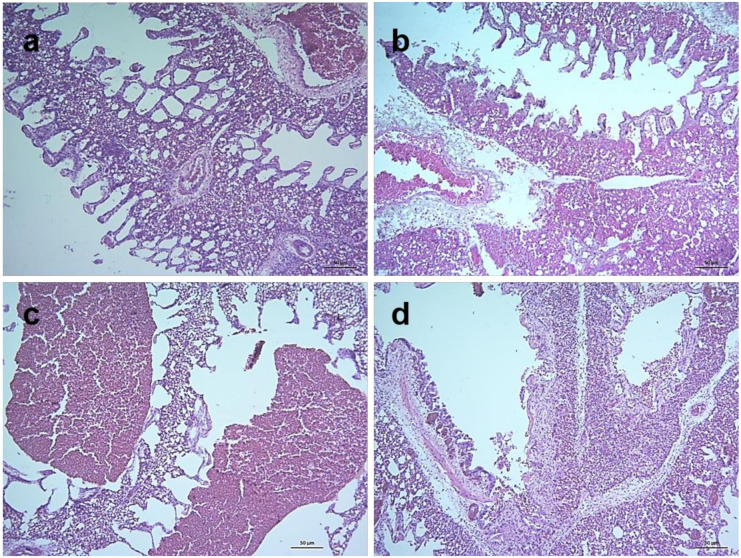
Histopathological changes in lungs of NDV infected chickens. Section of the lung of control chickens showing normal histology (**a**). Sections of lungs of infected chickens showing mild edema, congestion and hemorrhages at 24 hpi (**b**), edema, severe hemorrhages and collapsed and ruptured alveoli at 3 dpi (**c**), and mild congestion and hemorrhages, collapsed and ruptured alveoli with large number of inflammatory cells in the alveoli and interalveolar septa at 5 dpi (**d**). H&E stain, bar indicates magnification.

Histopathological changes of the control and NDV infected chickens were analyzed. The lesion in the lungs started with mild edema, congestion and hemorrhages at 24 hpi. These vascular changes were gradually associated with the rupture of alveoli as well as the infiltration of mononuclear cells. At 5 dpi infiltration of mononuclear cells became extensive along the collapsed and ruptured alveoli (Figure 6). In the trachea, edema and desquamation of lining epithelium were noticed at 2 dpi. Tracheitis with edema, desquamation of lining epithelium and infiltration of inflammatory cells were noticed at 3 dpi which progressed rapidly till the end of the study. Desquamated epithelium was found in the lumen of the trachea (Supplementary Figure S2).

**Figure 7:**
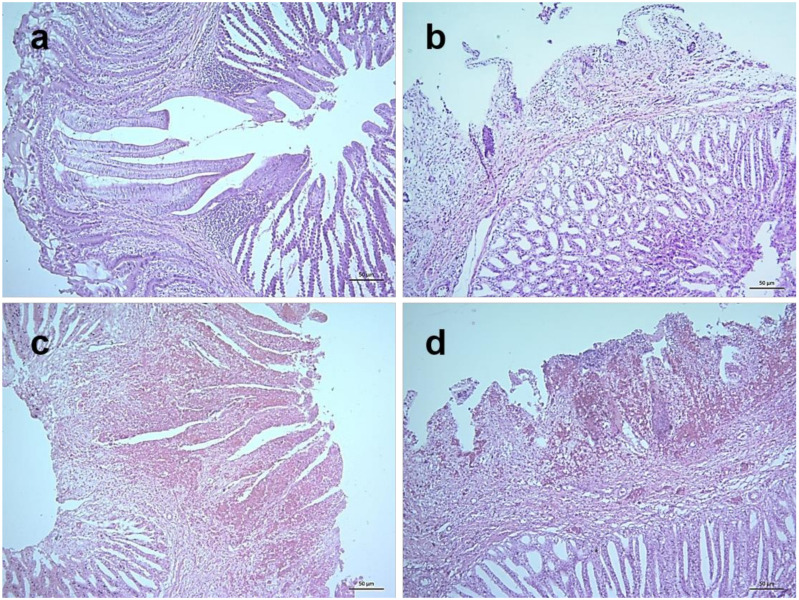
Histopathological changes in the proventriculus of NDV infected chickens. Section of the proventriculus of control chickens showing normal histology (**a**). Sections of the proventriculus of infected chickens showing slight congestion and edema at 3 dpi (**b**), severe hemorrhages in the finger-like mucosal folds (plicae), edema and mild infiltration of inflammatory cells at 4 dpi (**c**), and severe hemorrhages and necrosis in plicae, loss of lining epithelium, fusion and shortening of plicae and infiltration of large number of inflammatory cells at 5 dpi (**d**). H&E stain, bar indicates magnification.

In the alimentary canal, the proventriculus showed slight congestion and edema in the mucosa at 3 dpi followed by severe hemorrhages in the finger-like mucosal folds (plicae), edema and mild infiltration of inflammatory cells at 4 dpi. Severe hemorrhages and necrosis in plicae, loss of lining epithelium, fusion and shortening of plicae and infiltration of a large number of inflammatory cells were observed at 5 dpi (Figure 7). In the duodenum, there were mild hemorrhages in the lamina propria, shortening and fusion of villi, desquamation of lining epithelium and invasion of inflammatory cells at 4 dpi and similar but more severe lesions were observed at 5 dpi (Figure not shown).

The liver showed congestion in central veins at an early stage (3 dpi) followed by sinusoidal congestion with discrete hepatocellular degeneration at advanced stages (Supplementary Figure S3). Lesions in kidneys started with mild hemorrhages in renal tubules at 24 hpi followed by hemorrhages and nephrosis of renal tubules during 2–3 dpi. Severe congestion, hemorrhages and degenerative changes of renal tubules were observed after 4 dpi (Supplementary Figure S4).

**Figure 8:**
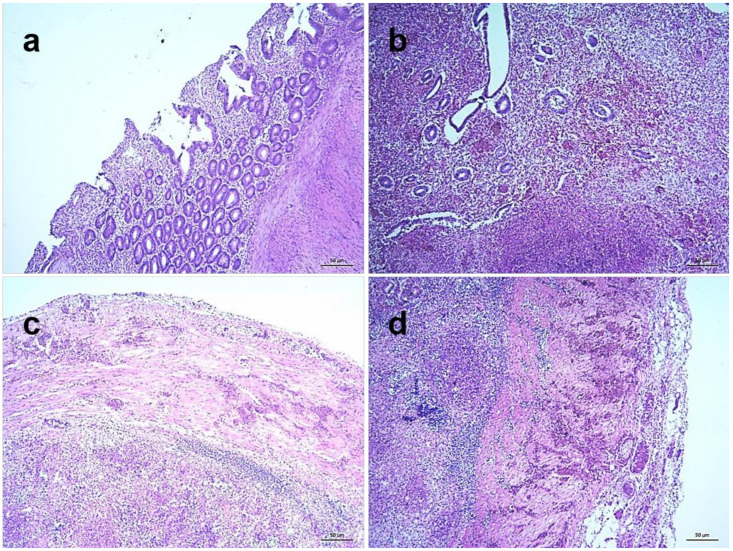
Histopathological changes in cecal tonsils of NDV infected chickens. Section of the cecal tonsil of control chickens showing normal histology (**a**). Sections of cecal tonsils of infected chickens showing hemorrhages in the lamina propria and associated lymphatic nodules at 3 dpi (**b**), mild hemorrhages and transmural invasion of inflammatory cells at 4 dpi (**c**) and severe hemorrhages with transmural invasion of large number of inflammatory cells at 5 dpi (**d**). H&E stain, bar indicates magnification.

**Figure 9:**
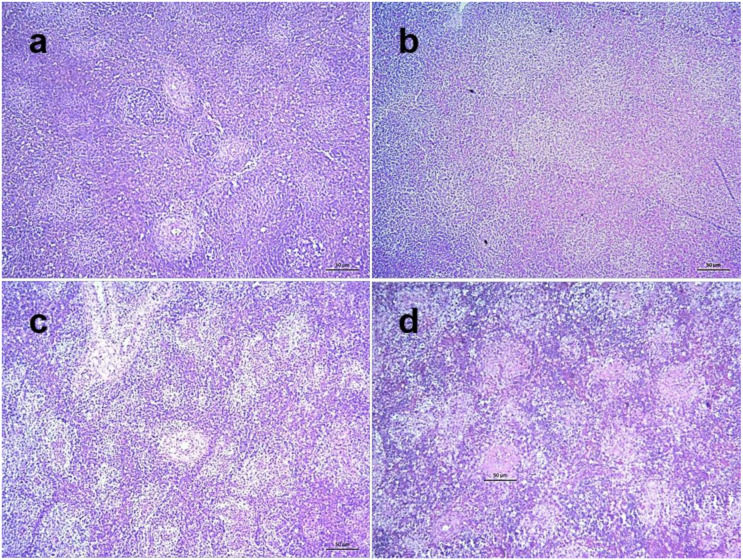
Histopathological changes in the spleen of NDV infected chickens. Section of the spleen of control chickens showing normal histology (**a**). Sections of spleens of NDV infected chickens showing normal architecture at 2 dpi (**b**), mild multifocal lymphoid depletion in the cortex of spleen at 3 dpi (**c**) and severe multifocal lymphoid depletion with increased number and sizes at 5 dpi (**d**). H&E stain, bar indicates magnification.

**Figure 10:**
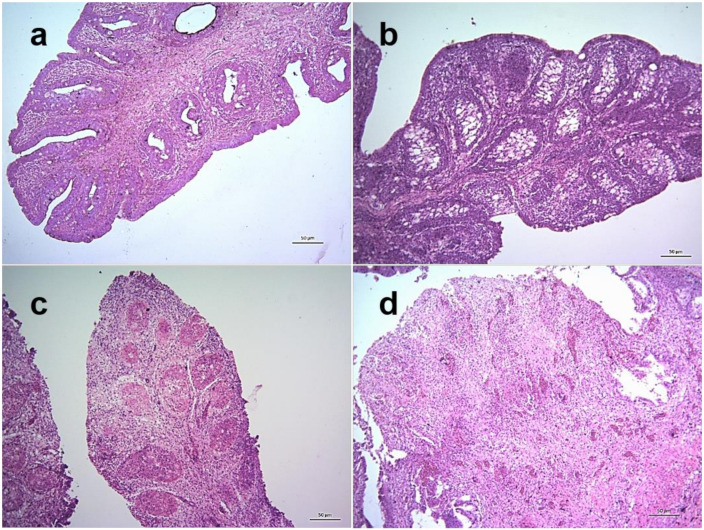
Histopathological changes in the bursa of Fabricius of NDV infected chickens. Section of the bursa of Fabricius of control chickens showing age-related mild lymphoid depletion (**a**). Sections of the bursa of Fabricius of infected chickens showing marked depletion of lymphocytes in the bursal follicle leaving numerous empty spaces at 24 hpi (**b**), follicular atrophy leaving necrotic masses, hemorrhages and infiltration of inflammatory cells at 4 dpi (**c**) and massive necrosis resulting in disappearance of lymphoid follicles, moderate hemorrhages and profuse inflammatory cells at 5 dpi (**d**). H&E stain, bar indicates magnification.

Among various lymphoid tissues and organs, cecal tonsils of NDV infected chickens showed hemorrhages in the lamina propria and associated lymphatic nodules at 3 dpi. Transmural invasion of inflammatory cells was found along with increased hemorrhages at 4 dpi and the severity further increased at 5 dpi (Figure 8). Marked lymphoid depletion was found in the spleen which started with small multiple depleted areas in the cortex at 3 dpi that progressed very rapidly with numerous focal lymphoid depletion of increased sizes during 4–5 dpi (Figure 9). Similarly, thymus showed moderate diffuse lymphoid depletion at 3 dpi followed by severe diffuse lymphoid depletion, hemorrhages and congestion and extensive necrosis during 4–5 dpi (Supplementary Figure S5). The bursa of Fabricius of control chickens showed some age-related mild lymphoid depletion. However, infected birds showed obvious lymphoid depletion marked by numerous empty spaces in the bursal follicles at 24 hpi. Necrosis in the bursal follicles, hemorrhages and infiltration of inflammatory cells followed by follicular atrophy were found during advance stages of the disease (Figure 10).

**Figure 11:**
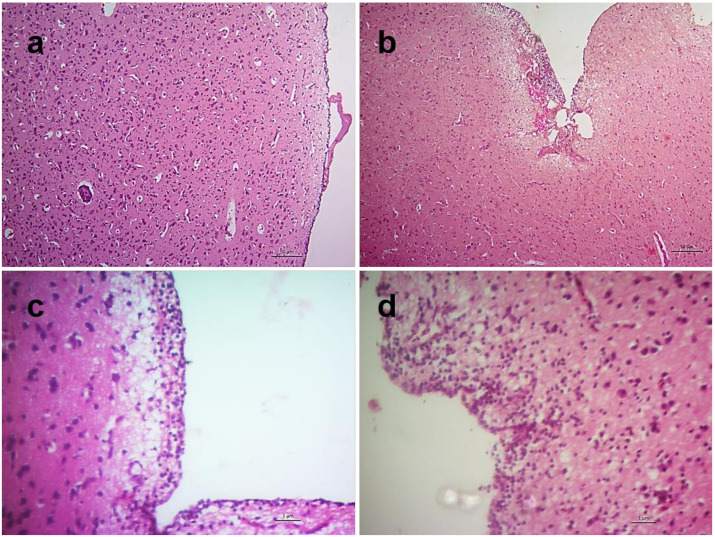
Histopathological changes in the brain of NDV infected chickens. Section of the brain of control chickens showing normal architecture (**a**). Sections of brains of infected chickens showing focal meningitis at 2 dpi (**b**), diffuse meningitis with encephalomalacia at 3 dpi (**c**) and diffuse meningoencephalitis with encephalomalacia at 5 dpi (**d**). H&E stain, bar indicates magnification.

Lesions in the brain started with focal meningitis at 2 dpi which turned into diffuse meningitis with encephalomalacia during 3–4 dpi. Diffuse meningoencephalitis with encephalomalacia was found later on at 5 dpi (Figure 11). Unless stated, sections of all tissues collected from control chickens showed normal histology of the studied organs.

### 2.4. Virus Detection in the Tissues by RT-PCR

NDV RNA was detected by RT-PCR in the lungs and bursa of Fabricius of the NDV infected dead chickens. Besides, bursal tissues were tested for the presence of IBDV RNA using RT-PCR and no IBDV RNA was detected in the bursa of Fabricius of the birds, ruling out the chance of co-infection.

## 3. Discussion

The Bangladeshi NDV strain BD-C161/2010 belonging to genotype XIII caused rapid mortality in chickens with signs of depression and paralysis. The pathological lesions were characterized by congestion, hemorrhages and edema in the lungs at the early stage, followed by degeneration and/or necrosis in different visceral organs, depletion of lymphocytes in lymphoid organs, infiltration of inflammatory cells in different visceral organs, and meningoencephalitis with encephalomalacia in the brain.

Strains of NDV are highly variable in their pathogenicity for chickens [4] and amino acid residues at the FPCS is postulated as the primary determinant of NDV virulence [13,17]. The HN protein with receptor recognition and neuraminidase activities of the virus determines and also contributes to virulence of NDV [18,19]. The isolate used in the present study, BD-C161/2010, possesses multiple basic amino acid residues (arginine or lysine) at FPCS with ^112^RRQKRF^117^ motif [12], suggestive of a velogenic pathotype [13]. Inoculation of the BD-C161/2010 isolate in 5-weeks old seronegative chickens also showed a relatively early commencement of clinical signs at 3 dpi and death at 4 dpi. The onset of clinical signs in NDV infected chickens could vary depending on the age and genetic endowment of the birds and dose and route of infection, genotype, host adaptability and pathogenicity of the virus [20]. For example, similar to our study, there are cases where clinical signs of velogenic NDV strains in chickens started at 2–3 dpi [21,22,23,24], whereas a variable onset of clinical signs between 2–16 dpi was also recorded [25]. An exact morbidity and mortality rate could not be calculated in this study as the infected chickens were sampled at different time intervals. However, four of the infected chickens died at each time point of 4 and 5 dpi, and one remaining bird was killed at moribund at 5 dpi. Our previous unpublished experiment using similar infection protocol with the same isolate produced 100% mortality by 8 dpi.

Respiratory lesion was observed very early at 24 hpi in the infected chickens indicating primary predilection of NDV to the respiratory system. Viruses attach to the respiratory epithelial cells by utilizing sialic acid receptor [26]. NDV antigen has been detected in lungs in other studies [27,28]. Virus replication in the lung induces regional immune response that would clear the virus in the subsequent period. Vascular changes like congestion, hemorrhages and edema were evident at a very early stage followed by alveolar damage and infiltration of mononuclear inflammatory cells at 3–5 dpi. Changes observed in the trachea of NDV infected chickens would also indicate possible primary replication of the virus in the upper respiratory tract, which would contribute to the development of the immune responses [27,28].

The NDV infected chickens showed hemorrhagic and necrotizing lesions in the gastrointestinal tract, particularly at gut-associated lymphoid tissues (GALT), starting at 3 dpi and progressed very rapidly, which is typical of velogenic viscerotropic NDV [7,21,29,30,31]. Hemorrhages and edema in the mucosa of the proventriculus and intestine were observed at 3 dpi followed by necrosis, fusion of mucosal folds and infiltration of mononuclear inflammatory cells later on. Similar changes in the proventriculus and intestine of chickens infected with velogenic strains of NDV were reported with a slightly delayed onset (at 4 dpi) of the changes [22,29,32]. Extensive replication of the velogenic NDV has been described in the digestive tract [7,27]. Following primary viremia, NDV reaches different visceral organs including liver and kidneys through blood circulation causing secondary viremia [26]. Virus replication in the liver and kidney may cause vascular injury leading to congestion and hemorrhages at an early stage followed by discrete hepatocellular degeneration and renal tubular nephrosis during advanced stage [7,27]. However, marked hepatocellular necrosis in the liver and necrotizing interstitial nephritis in the kidney along with lymphocytic infiltration were described in the NDV infected chickens that survived a longer duration (8–10 dpi) [24].

The NDV strain BD-C161/2010 produced extensive necrosis of lymphoid tissues including the thymus, spleen, bursa of Fabricius, cecal tonsils and GALT as described earlier [7,21,29,30,31]. Extensive replication of the virus in lymphoid tissues and apoptosis could lead to severe lymphoid depletion [5,7,21,29,30,33,34]. Among various lymphoid tissues, cecal tonsils and GALT showed hemorrhagic and necrotic lesions whereas marked lymphoid depletion with necrosis and atrophy of the organ was found in the thymus, spleen and bursa of Fabricius. Lesions in most of the lymphoid organs started at 3 dpi except in cecal tonsils and bursa of Fabricius, which showed changes starting at 24 hpi. Hemorrhages in the cecal tonsils were shown to commence at an early time point (2 dpi) followed by severe hemorrhages and necrosis at 4–5 dpi [21,32]. However, the early hemorrhagic changes in cecal tonsils at 24 hpi could be due to initial replication of the virus and entry into systemic circulation through cecal tonsils following nasopharyngeal inoculation. Severe lymphoid depletion and necrosis in the lymphoid organs (thymus, bursa of Fabricius and spleen) of chickens infected with velogenic viscerotropic strains of NDV were mostly described at 4–5 dpi [21,35] as compared to the early commencement at 2–3 dpi in the present study. Such differences could be due to greater virulence and lymphotropic nature of the isolate. However, the contribution of co-infecting immunosuppressive viral diseases like IBDV has been ruled out by RT-PCR.

Neuron and glial cells permit productive replication of virulent NDV and may cause neuronal damages and inflammatory responses [5,36,37]. We have also noticed an early and mild focal meningitis along with encephalomalacia at 2 dpi reflecting in clinical signs like depression at 3 dpi that turned into moderate and diffuse meningoencephalitis, possibly leading to dropped wings and paralysis at 5 dpi. Profound lesions in the brain such as non-purulent encephalitis, perivascular cuffing, loss of Purkinje cells, etc., were reported in NDV infected chickens that survived longer (usually more than 5 dpi) [3,5,6,21]. In a separate unpublished study, the present isolate also showed a profound neurological involvement in infected chickens that survived until 8 dpi. The neuropathogenicity of the isolate in chickens should be studied using a lower dose of the inoculum in experimental infection.

In conclusion, the genotype XIII NDV strain BD-C161/2010 infection in chickens produced acute infection with severe pneumonia at an early stage, followed by profound necrotizing and hemorrhagic lesions in the digestive system, lymphoid depletion and modest neuronal changes at an advance stage. The clinicopathological features of the Bangladeshi genotype XIII NDV strain BD-C161/2010 confirm its velogenic viscerotropic nature. The present pathomorphological study should be followed by a molecular pathogenesis study with the analysis of early cytokine responses and tissue distribution of the virus in chickens experimentally infected with the genotype XIII NDV strain.

## 4. Materials and Methods

### 4.1. Ethics Statement

All applicable national and institutional guidelines for the care and use of animals were followed. The study was carried out in accordance with the recommendation of the Ethical Standard of Research Committee of Bangladesh Agricultural University, Mymensingh. The protocol and procedures employed were reviewed and approved by the Ethical Standard of Research Committee (Ref. No. BAURES/ESRC/693/2020; Dated: 10.06.2020).

### 4.2. Inoculum Preparation

For pathogenesis study, a Bangladeshi strain (BD-C161/2010) of NDV was retrieved from the virus repository of the Department of Pathology, Bangladesh Agricultural University. The virus was originally isolated in embryonated chicken eggs from a natural outbreak in chickens in 2010 [10]. Later, the isolate has been characterized as a velogenic pathotype and classified under genotype XIII based on polybasic FPCS motif and phylogenetic analysis, respectively [12]. The virus was propagated in 9 days old specific pathogen-free (SPF) eggs via allantoic cavity route and the infected allantoic fluid was harvested. The identity of the virus in the infected allantoic fluid was confirmed by RT-PCR as described previously [12,38]. Besides, the inoculum was tested for avian influenza (AI), infectious bronchitis virus (IBV) and infectious bursal disease virus (IBDV) using specific RT-PCR as described elsewhere to rule out the chance of contamination and found negative [39,40,41]. The allantoic fluid was titrated in embryonated chicken eggs in terms of embryo infectious dose 50 (EID_50_).

### 4.3. Experimental Infection

A total of 30 layer chicks of ISA Brown strain were raised from day-old in relative isolation with food and water ad libitum. At 35 days, blood samples were collected randomly from 10 chicks to check the level of maternally derived antibody by hemagglutination inhibition (HI) test. Most of the tested sera showed very low (≤16 HI titre) or no maternal antibody titers. At 35 days of age, chickens were divided into two groups, infected (n = 24) and control (n = 6) and housed separately. Chickens from the infected group were inoculated through intranasal and intraocular routes with 10^5^ EID_50_ (0.2 mL/bird) of the virus. The control birds received 0.2 mL of uninfected allantoic fluid via the same routes. Chickens were monitored twice daily for clinical signs, morbidity and mortality.

### 4.4. Autopsy and Sample Collection

Three chickens from the infected group were killed at each time points of 24, 48 and 72 h post-infection (hpi) and subjected to detailed examination at autopsy. At 96 and 120 hpi, freshly dead or moribund chickens were examined at autopsy. Besides, two chickens from the control group were examined at each time points of 24, 72 and 120 hpi. Pathological changes in different organs were recorded in detail. Tissues from the lungs and bursa of Fabricius of NDV infected dead chickens were collected aseptically in sterile tubes for virus detection. In addition, tissues from the lungs, trachea, cecal tonsils, proventriculus, intestine, liver, kidneys, spleen, thymus, bursa of Fabricius, and brain from both control and infected chickens at different time points were collected in 10% neutral buffered formalin for histopathology.

### 4.5. Detection of Viral RNA in Tissues

The presence of NDV RNA in tissues of the infected chickens was detected as proof-of-the infection. Besides, the presence of IBDV RNA in tissues was also tested to rule out the co-infection. Briefly, 20% of tissue homogenates were prepared from the collected lungs and bursa of Fabricius. RNA was extracted using the PureLink™ RNA Mini Kit (ThermoFisher Scientific, Waltham, MA, USA). The confirmation of the infection in the infected chickens was performed by detecting the NDV in tissues by RT-PCR [12,38] using SuperScript^®^ III One-Step RT-PCR System with Platinum^®^ Taq DNA polymerase (Life Technologies, Carlsbad, CA, USA). Besides, bursal tissues were tested for the presence of IBDV using RT-PCR as described previously to rule out the chance of co-infection [39].

### 4.6. Histopathology

Formalin-fixed tissues were processed and embedded with paraffin. A 5 µm section was prepared from the paraffin block and then stained with routine hematoxylin and eosin (H&E) following the standard procedure. The slides were examined under photomicroscope (ZEISS Primo Star) and the images were recorded electronically.

## Supplementary Materials

The following are available online at https://www.mdpi.com/2076-0817/9/7/539/s1. Supplementary Figure S1: Gross pathological lesions in the liver of NDV infected chickens. The liver of control chickens showing normal appearance (a). Livers of infected chickens showing mild congestion and hemorrhages at 3 dpi (b), hemorrhages and congestion with increased intensity and enlargement of the organ at 4 dpi (c) and 5 dpi (d). Supplementary Figure S2: Histopathological changes in the trachea of NDV infected chickens. Section of the trachea of control chickens showing normal architecture (a). Sections of tracheas of infected chickens showing edema and desquamation of lining epithelium at 2 dpi (b), edema, desquamation of lining epithelium and mild infiltration of inflammatory cells at 3 dpi (c) and congestion, desquamation of lining epithelium and moderate infiltration of inflammatory cells at 5 dpi (d). H&E stain, bar indicates magnification. Supplementary Figure S3: Histopathological changes in the liver of NDV infected chickens. Section of the liver of control chickens showing normal architecture (a). Sections of livers of infected chickens showing congestion in central vein at 3 dpi (b), sinusoidal congestion with discrete hepatocellular degeneration at 4 dpi (c) and severe congestion and hemorrhages with hepatocellular degeneration at 5 dpi (d). H&E stain, bar indicates magnification. Supplementary Figure S4: Histopathological changes in the kidney of NDV infected chickens. Section of the kidney of control chickens showing normal histology (a). Sections of kidneys of infected chickens showing congestion and mild degenerative changes of renal tubules at 3 dpi (b), moderate hemorrhages, congestion and degenerative changes of renal tubules at 4 dpi (c) and severe hemorrhages, congestion and degenerative changes of renal tubules at 5 dpi (d). H&E stain, bar indicates magnification. Supplementary Figure S5: Histopathological changes in the thymus of NDV infected chickens. Section of the thymus of control chickens showing normal histology (a). Sections of the thymus of infected chickens showing moderate lymphoid depletion with necrosis at 3 dpi (b), severe lymphoid depletion, extensive necrosis, hemorrhages and congestion at 4 dpi (c) and 5 dpi (d). H&E stain, bar indicates magnification.
