# Supplementary material for: Sequential Pathology of a Genotype XIII Newcastle Disease Virus from Bangladesh in Chickens on Experimental Infection
Source: Pathogens. 2020 Jul 6;9(7):539. doi: 10.3390/pathogens9070539 (PMC7399908; doi:10.3390/pathogens9070539)
Supplement: Supplementary file 1 [file pathogens-09-00539-s001.pdf]

Supplementary Materials

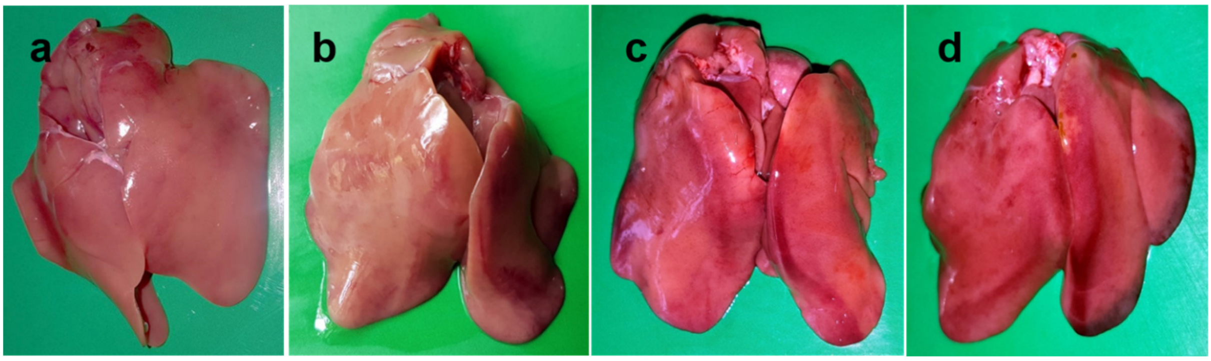

Figure S1. Gross pathological lesions in the liver of NDV infected chickens. The liver of control chickens showing normal appearance (a). Livers of infected chickens showing mild congestion and hemorrhages at 3 dpi (b), hemorrhages and congestion with increased intensity and enlargement of the organ at 4 dpi (c) and 5 dpi (d).

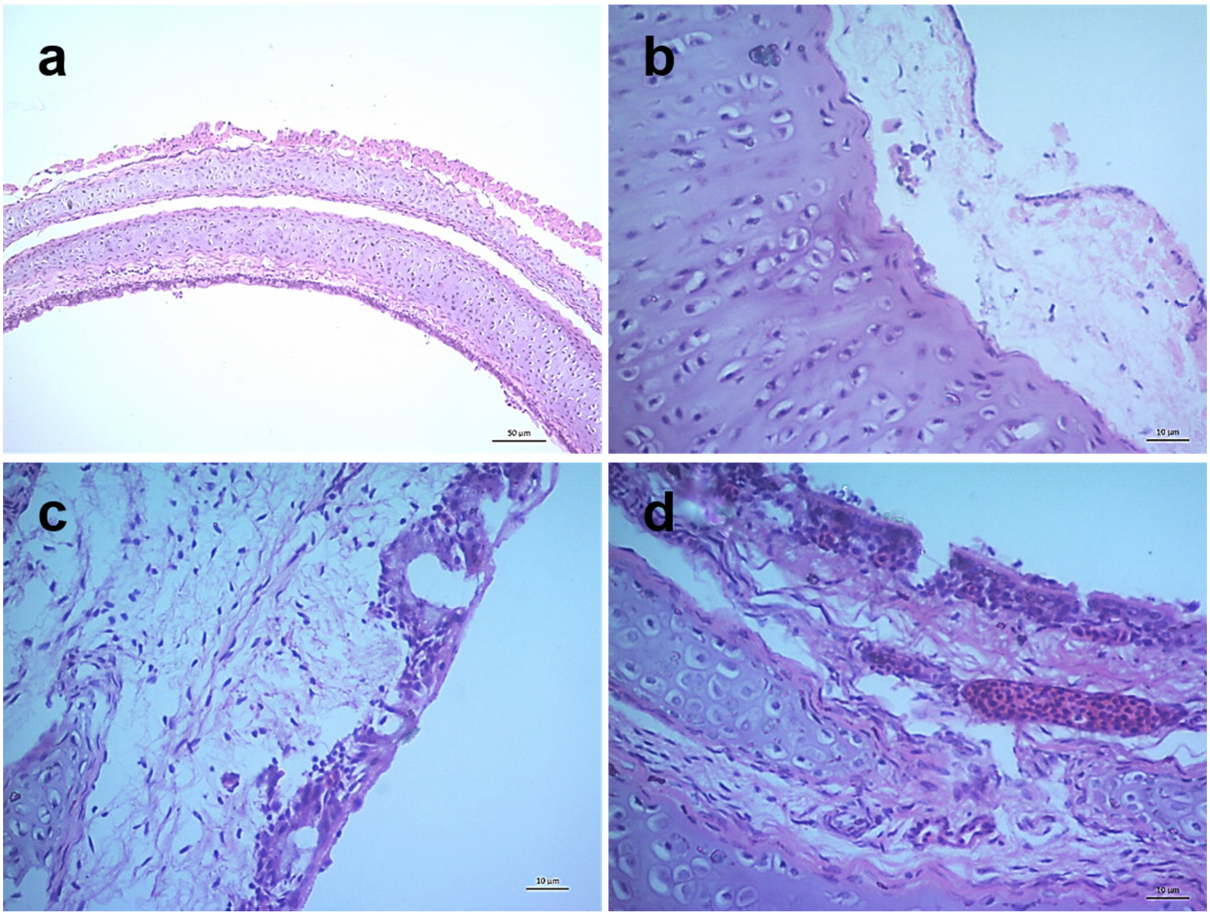

Figure S2. Histopathological changes in the trachea of NDV infected chickens. Section of the trachea of control chickens showing normal architecture (a). Sections of tracheas of infected chickens showing edema and desquamation of lining epithelium at 2 dpi (b), edema, desquamation of lining epithelium and mild infiltration of inflammatory cells at 3 dpi (c) and congestion, desquamation of lining epithelium and moderate infiltration of inflammatory cells at 5 dpi (d). H&E stain, bar indicates magnification.

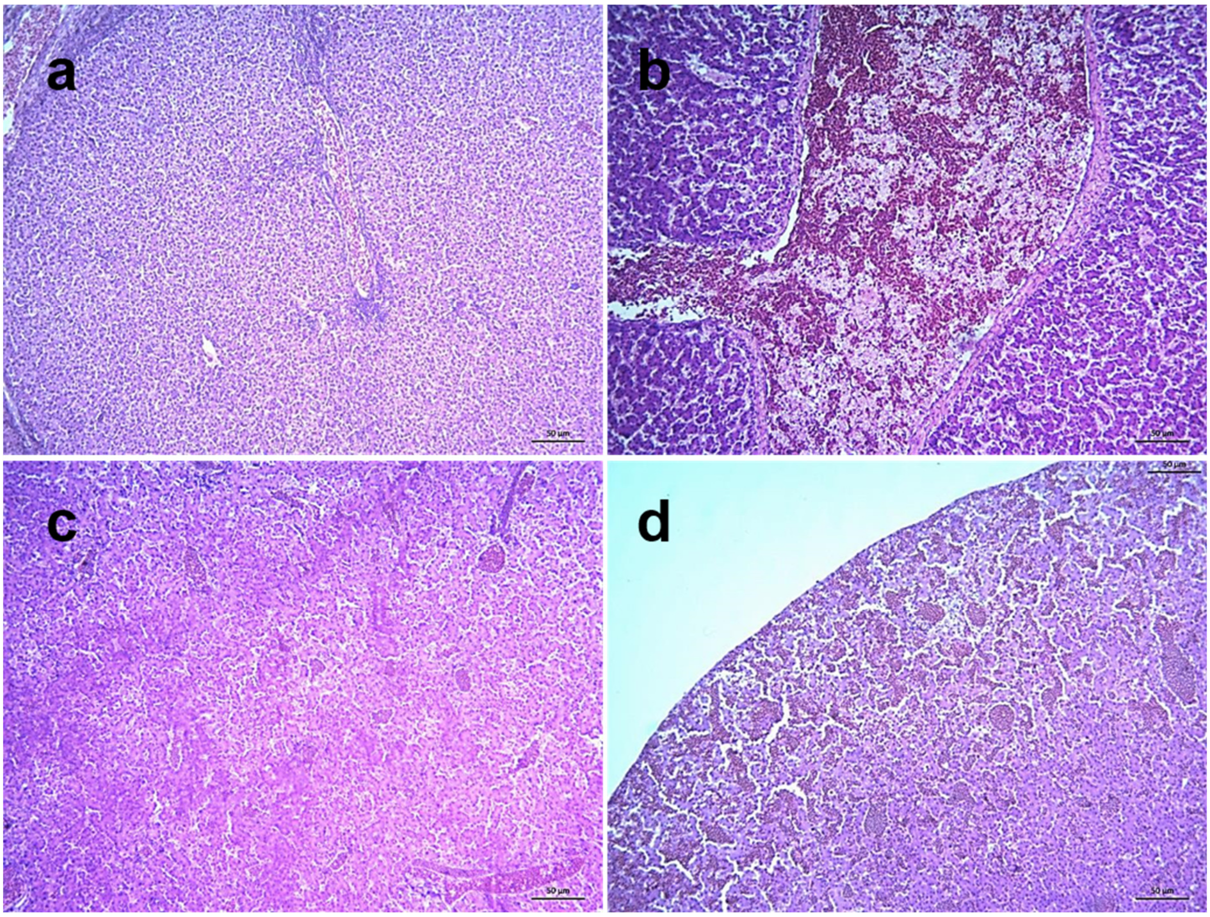

Figure S3. Histopathological changes in the liver of NDV infected chickens. Section of the liver of control chickens showing normal architecture (a). Sections of livers of infected chickens showing congestion in central vein at 3 dpi (b), sinusoidal congestion with discrete hepatocellular degeneration at 4 dpi (c) and severe congestion and hemorrhages with hepatocellular degeneration at 5 dpi (d). H&E stain, bar indicates magnification.

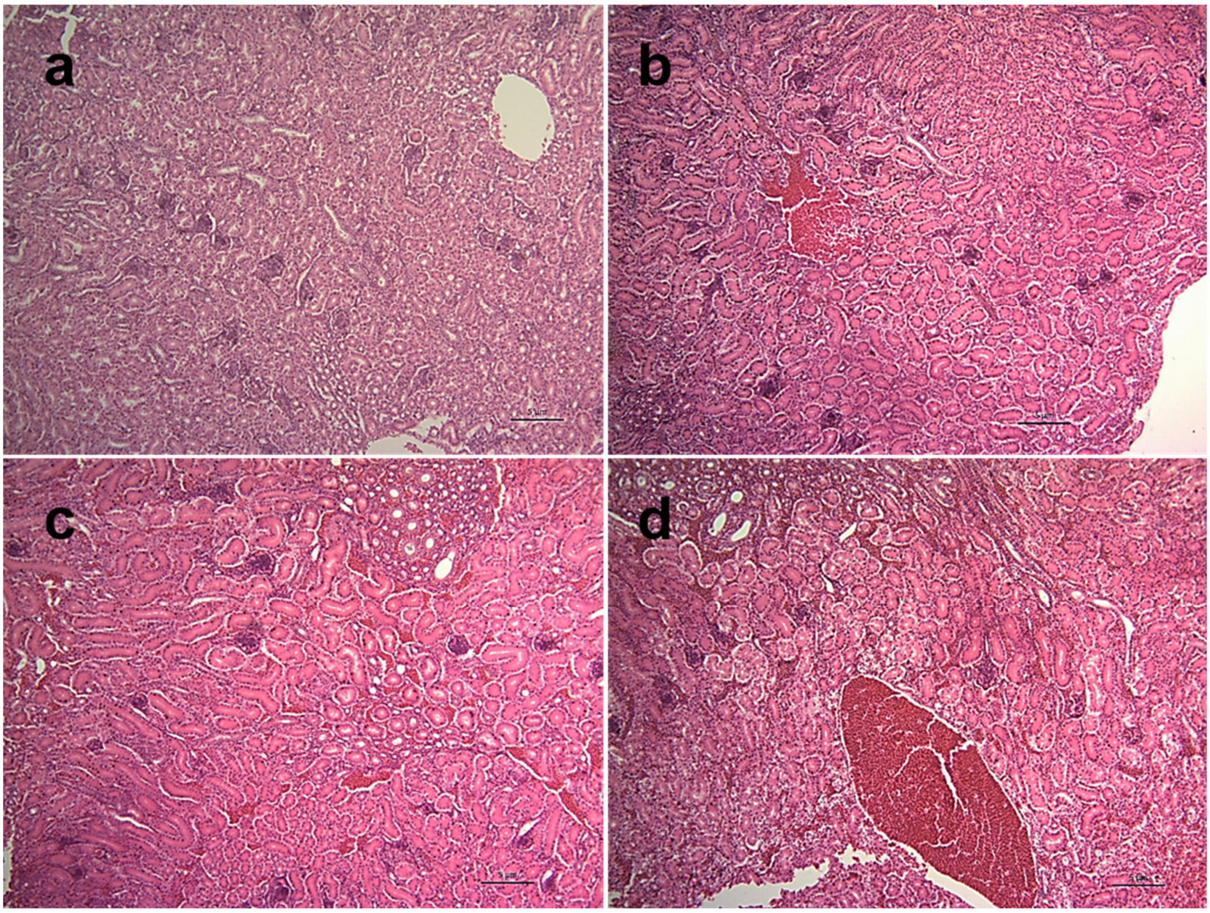

Figure S4. Histopathological changes in the kidney of NDV infected chickens. Section of the kidney of control chickens showing normal histology (a). Sections of kidneys of infected chickens showing congestion and mild degenerative changes of renal tubules at 3 dpi (b), moderate hemorrhages, congestion and degenerative changes of renal tubules at 4 dpi (c) and severe hemorrhages, congestion and degenerative changes of renal tubules at 5 dpi (d). H&E stain, bar indicates magnification.

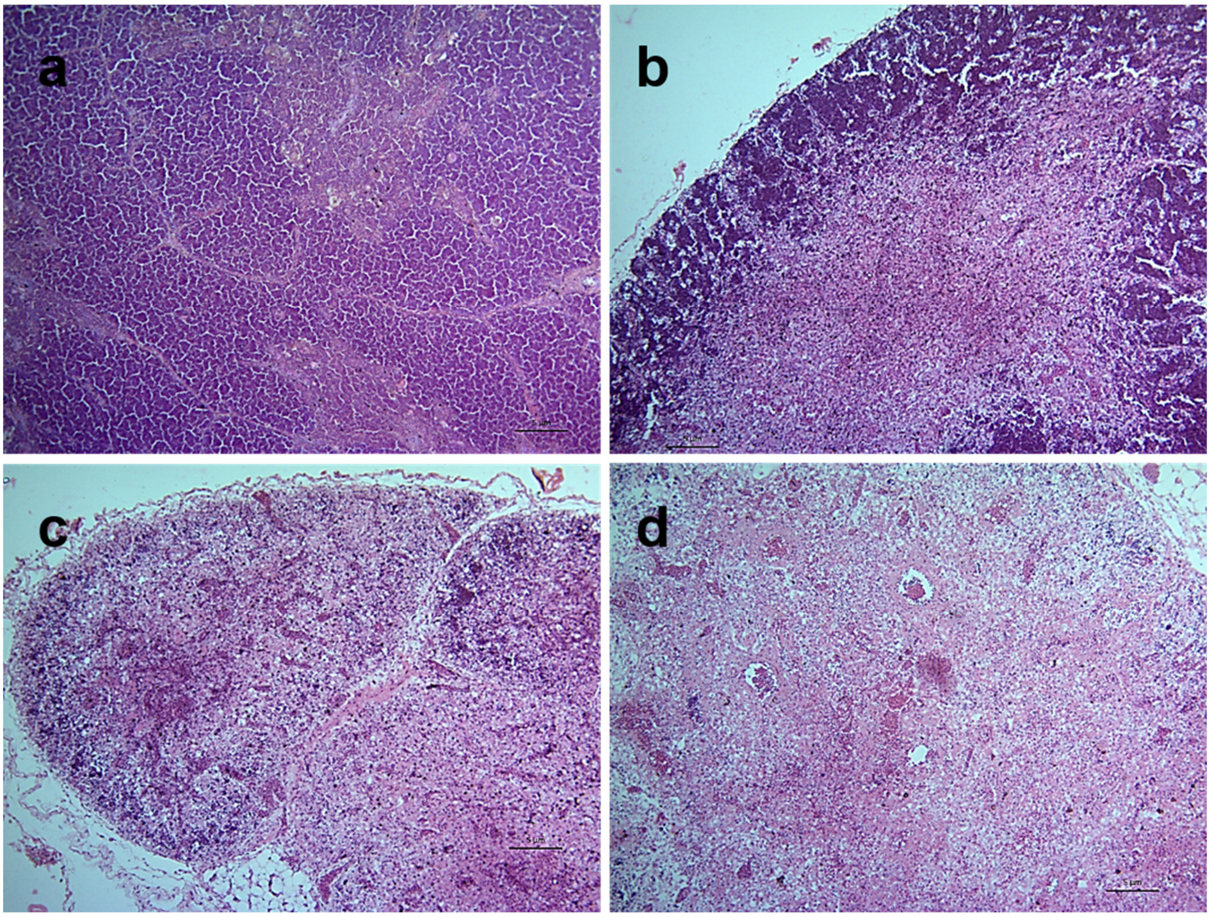

Figure S5. Histopathological changes in the thymus of NDV infected chickens. Section of the thymus of control chickens showing normal histology (a). Sections of the thymus of infected chickens showing moderate lymphoid depletion with necrosis at 3 dpi (b), severe lymphoid depletion, extensive necrosis, hemorrhages and congestion at 4 dpi (c) and 5 dpi (d). H&E stain, bar indicates magnification.
